# Global Identification and Systematic Analysis of Lysine Malonylation in Maize (*Zea mays* L.)

**DOI:** 10.3389/fpls.2021.728338

**Published:** 2021-08-20

**Authors:** Min Xu, Xiaomin Tian, Tingting Ku, Guangyuan Wang, Enying Zhang

**Affiliations:** ^1^College of Agronomy, Qingdao Agricultural University, Qingdao, China; ^2^Shandong Province Key Laboratory of Applied Mycology, College of Life Sciences, Qingdao Agricultural University, Qingdao, China

**Keywords:** *Zea mays* L., malonylome, photosynthesis, Calvin cycle, post-translational modification

## Abstract

Lysine malonylation is a kind of post-translational modifications (PTMs) discovered in recent years, which plays an important regulatory role in plants. Maize (*Zea mays* L.) is a major global cereal crop. Immunoblotting revealed that maize was rich in malonylated proteins. We therefore performed a qualitative malonylome analysis to globally identify malonylated proteins in maize. In total, 1,722 uniquely malonylated lysine residues were obtained in 810 proteins. The modified proteins were involved in various biological processes such as photosynthesis, ribosome and oxidative phosphorylation. Notably, a large proportion of the modified proteins (45%) were located in chloroplast. Further functional analysis revealed that 30 proteins in photosynthesis and 15 key enzymes in the Calvin cycle were malonylated, suggesting an indispensable regulatory role of malonylation in photosynthesis and carbon fixation. This work represents the first comprehensive survey of malonylome in maize and provides an important resource for exploring the function of lysine malonylation in physiological regulation of maize.

## Introduction

Post-translational protein modification (PTM) plays an important role in the regulation of various cellular processes, which often occurs during or after protein biosynthesis. PTM regulates protein functions by introducing new functional groups such as acetyl, phosphorus, methyl, acetyl, ubiquitin and malonyl groups (Zhang et al., 2016, 2017; Liu et al., 2018). By reversibly adding functional groups to proteins, PTM regulates a variety of biochemical metabolisms, including intracellular protein localization, enzyme activity and protein stability in cells (Olsen et al., 2006; Schwickart et al., 2010; Song et al., 2016). In plants, PTMs diversify protein behaviors, thus increasing the functionality of the proteome (Ma et al., 2021). In *Arabidopsis*, an ubiquitylome analysis revealed that the ubiquitin-proteasome system plays a central role in the regulation of plant innate immunity (Ma et al., 2021). MPK4, a stress-responsive protein kinase of *Arabidopsis*, has been demonstrated to be activated by phosphorylation and oxidation (Zhang et al., 2015, 2019). These studies indicate that many types of PTMs, including ubiquitylation and phosphorylation, are involved in the regulation of physiological processes in plants.

Among these PTMs mentioned above, lysine malonylation is a new PTM recently discovered, which plays an important regulatory role in cell metabolisms (Hirschey and Zhao, 2015). In malonyl modification, the positively charged lysine of protein is chemically modified by the addition of a negatively charged malonyl group and this charge change has been found to play an important role in metabolic regulation and protein function (Taherzadeh et al., 2018). Previous studies have proved that malonyl-CoA might be one of donors for malonyl groups (Peng et al., 2011; Colak et al., 2015). However, the enzyme that regulates the malonylation status of proteins is still unclear (Liu et al., 2018). It was found that Sirt5, one of lysine deacetylases (KDACs) could reduce the level of malonylation of proteins in mammals (Peng et al., 2011; Colak et al., 2015; Nishida et al., 2015). Other types of acylations, including glutarylation and succinylation, are also found to be regulated by sirtuin-class deacylases (Choudhary et al., 2014). Therefore, it is speculated that similar to protein acetylation modification, malonyl modification is also regulated by KDAC and lysine acetyltransferase (KAT) (Liu et al., 2018). Many studies have shown that histone proteins undergo malonyl modification in both prokaryotes and eukaryotes (Colak et al., 2015; Liu et al., 2018; Nie et al., 2020), which may be involved in regulating gene transcription. In addition to histone proteins, a large number of other proteins in the mitochondria, chloroplasts, cytoplasm and nucleus have also been discovered to be modified by malonyl modification, suggesting that protein malonylation is associated with a variety of biological metabolisms (Liu et al., 2018).

Thanks to the advance of liquid chromatography mass spectrometry (LC-MS/MS) and the development of immunoprecipitation of lysine malonylated peptides, lysine malonylation can be studied at a proteomic level. As a result, large amounts of lysine malonylated proteins were obtained. Bioinformatics analysis showed that these malonylated proteins identified in bacteria (Qian et al., 2016; Xu et al., 2016; Fan et al., 2017; Ma et al., 2017), plants (Liu et al., 2018; Mujahid et al., 2018), and mammals (Peng et al., 2011; Colak et al., 2015) are associated with multiple cellular functions including cell growth and apoptosis, energy synthesis, and transcriptional regulation. However, compared with these species, the malonylome of maize is little studied. Annotation of malonylation in proteomics is therefore the first-crucial step to decipher its potential in physiological roles of maize.

Maize (*Zea mays* L.), one of the most widely cultivated cereal crops in the world, is a staple food for many people and an important source of nutrients for animal feed (Yang et al., 2014). Qingnong-11 is a semi-compact maize variety featured by its high yield and wide adaptability, and is widely planted in Shandong Province, China. There are a large number of acyltransferase genes and deacylase genes in maize genome. Therefore, we speculate that lysine malonylation may play a critical role in maize metabolism and development. To confirm this hypothesis, we conducted a malonylome study in maize using the combination of high-resolution LC-MS/MS analysis coupled with highly sensitive immune-affinity purification. Finally, we identified 1,722 malonylated sites in 810 unique proteins controlling multiple biological processes in maize variety Qingnong-11. All the identified proteins were further analyzed by bioinformatics. It was found that the identified proteins were involved in a variety of cellular functions and biological processes. Importantly, 45% identified proteins were found to locate in the chloroplast and further analysis revealed that a total of 30 malonylated proteins were involved in photosynthesis suggesting an important role of lysine malonylation in photosynthesis of maize. The obtained results provided a system-wide view of maize malonylome and an affluent dataset for investigating the physiological functions of protein malonylation in maize and possibly all plants.

## Materials and Methods

### Plant Materials

Qingnong-11, a Maize (*Z. mays* L.) variety used in the study, was obtained by crossing L1786 as female parent with L1111 as male parent, which was bred by Maize Breeding Team of Qingdao Agricultural University. Qingnong-11 has been widely cultivated in Shandong Province, China. The cultivar has been approved by the Shandong Crop Variety Appraisal Committee (Number: 2015001). Qingnong-11 was grown in a greenhouse at 25°C with 60% relative humidity, 16 h light (5000-lux)/8 h dark and the CO_2_ level in the greenhouse was 300–400 ppm. Young maize leaves were collected at the four-leaf stage for total protein extraction.

### Total Protein Extraction From Maize and Trypsin Digestion

Total protein was extracted from the young leaves according to the methods described (Zhang et al., 2016; Liu et al., 2018). First, the samples from three independent biological experiments were combined, where each sample (0.8 g) consists of 2 leaves from one plant. The total leaves (2.4 g) were then grinded in liquid nitrogen. The resulting cellular powder was mixed with protein-extraction buffer (8 M urea, 10 mM dithiothreitol, 1% triton-100, and 1% protease inhibitor cocktail). After sonication 3 times on ice using an ultrasonic processor (650E, SCIENTZ Biotech) with 35 pulses at 30% amplitude, where each pulse consists of 3 s of sonication followed by a 5 s rest-period, the remaining cell-debris was removed at 20,000 × g 4°C for 10 min and the resulting supernatant was further mixed with cold 20% trichloroacetic acid for protein precipitation. The extracted protein was redissolved in 8 M urea containing 100 mM NH_4_HCO_3_ and the protein concentration was determined using a BCA protein assay kit (Beyotime Biotechnology) according to the manufacturer’s instructions. For digestion, the protein solution was reduced in 10 mM dithiothreitol followed alkylated in 20 mM iodoacetamide (Zhang et al., 2016). Afterward, the protein was digested by trypsin as described (Liu et al., 2018).

### Affinity Enrichment of Modified Peptides

The digested protein in above section was separated with a gradient of 8 to 32% acetonitrile (pH 9.0) over 60 min into 60 fractions using high pH reverse phase HPLC equipped with a Thermo Betasil C18 column (5 μm particles, 10 mm ID, 250 mm length). Then, the peptides were combined into 4 fractions and dried by vacuum centrifuging. For enrichment, tryptic peptides were dissolved in NETN buffer (100 mM NaCl, 1 mM EDTA, 50 mM Tris–HCl, 0.5% NP-40, pH 8.0) followed by incubation with pre-washed anti-malonyllysine antibody conjugated agarose beads (PTM-904, PTM Bio) at 4°C for 12 h. The enriched peptides from the antibody beads were eluted using 0.1% trifluoroacetic acid followed by cleaning up using C18 ZipTips (Millipore) as described (Zhang et al., 2016; Liu et al., 2018).

### LC-MS/MS Analysis

First, an EASY-nLC 1,000 ultra-performance liquid chromatography (UPLC) system (ThermoFisher) was employed to separate the tryptic peptides (Wang et al., 2019). The separated peptides were then analyzed by tandem MS/MS in Q Exactive^TM^ Plus (ThermoFisher) (Liu et al., 2018). The normalized collision energy (NCE) was set as 28 and the electrospray voltage applied was set as 2.0 kV. The m/z scan range was 350 to 1,800 for full scan. The intact peptides and the fragments were detected in the Orbitrap at resolutions of 70,000 and 17,500, respectively. A data-dependent process in which 1 MS scan was followed by 20 MS/MS scans and the dynamic exclusion was 15.0 s (Wang et al., 2019). The automatic gain control (AGC) and fixed first mass were set at 5E4 and 100 m/z, respectively.

### Database Search

Maxquant search engine (v.1.5.2.8,^1^) was employed to process the resulting mass spectra dataset. The obtained MS/MS data were searched against UniProt-*Z*. *mays* database (99,368 sequences,^2^, taxonomy ID: 4577, download at Nov 5, 2018) concatenated with reverse decoy database. Trypsin/P was designated as lyase and up to four missing cleavages were allowed. For precursor ions, the mass tolerance was set as 20 ppm in the first search and 5 ppm in the main search (Zhang et al., 2016). For fragment ions, the mass tolerance was set as 0.02 Da. The fixed modification was specified as carbamidomethyl on cysteine and the variable modifications were specified as oxidation on methionine, acetylation on protein n-terminal, and malonylation on lysine. The false discovery rate (FDR) was adjusted to < 1% and minimum score for modified peptides was set > 40 (Liu et al., 2018).

### Bioinformatics Analysis

The obtained malonylome was annotated using Gene Ontology (GO) from UniProt-GOA database as described (Fang et al., 2018). InterProScan was employed to analyze domain functional description of the modified protein (Wang et al., 2019). The metabolic pathway involved in the modified proteins were analyzed by Kyoto Encyclopedia of Genes and Genomes (KEGG) database (Nie et al., 2020). If *p*-value was less than 0.05 in each cluster item of GO, KEGG, and protein domain, it was considered significant (Wang et al., 2019). The conserved motifs in the malonylated proteins which constituted with amino acid in specific positions were obtained by software MoMo (Motif-X algorithm) (V5.0.2) (Chou and Schwartz, 2011). NetSurfP was used to process secondary structure description of the malonylated proteins (Klausen et al., 2019). For the subcellular localization prediction of the malonylated proteins, software Wolfpsort (PSORT/PSORT II) was employed as described (Horton et al., 2007). The conservation of identified proteins between maize and other species was performed by BLASTP (Liu et al., 2018). To obtain the protein-protein interaction (PPI) network of the modified proteins, all the malonylated proteins were searched against STRING database (v. 11.0) and the networks obtained were visualized by software Cytoscape (Shannon et al., 2003; Szklarczyk et al., 2015).

### Immunoprecipitation and Immunoblot Analysis

The leaves of maize were ground in liquid nitrogen and the proteins were then purified as described (Liu et al., 2018). Briefly, the proteins (20 μg) of maize were electrophoresed on 12% gel in SDS-PAGE for 2.5 h with voltage 110 V followed by electrotransfer onto a polyvinylidene difluoride (PVDF) membrane with constant current 300 mA for 1 h. After blocking in 5% skim milk powder in TBST buffer (20 mM Tris–HCl, 150 mM NaCl, 0.05% Tween 20) for 1 h, the PVDF membrane was first incubated with anti-malonyllysine mouse mAb (1:1,000 dilution, PTM-902, PTM Biolabs) at 4°C for 12 h. Anti-mouse IgG peroxidase conjugated secondary antibody (A9044, Sigma) was used at a 1:10,000 dilution, and incubation time was 2 h at room temperature. The signals were detected using an enhanced chemiluminescence (ECL) immunoblotting detection kit (Beyotime Biotechnology).

## Results

### Identification and Analysis of Lysine Malonylated Proteins in Maize

**FIGURE 1:**
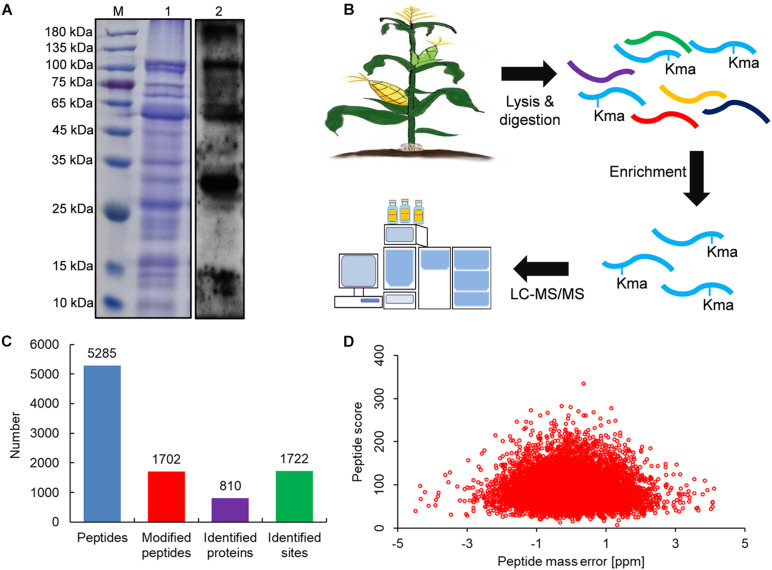
Global identification of lysine malonylation sites in maize. **(A)** SDS-PAGE (1) and signal in immunoblotting with antimalonyllysine antibody (2). **(B)** Work procedure used in this study. **(C)** Number of malonylated proteins and sites in maize. **(D)** Distribution of peptide mass error.

To investigate whether maize contains lysine malonylated proteins, we first performed an immunoblotting analysis with anti-malonyllysine antibody. As shown in Figure 1A, a variety of immunoblot signals with different molecular weights were obtained, suggesting that the malonylated proteins were abundant in maize. We further performed a proteome analysis of lysine malonylated proteins in maize as the procedures shown in Figure 1B. Finally, 5285 peptides were obtained by mass spectrometry, of which 1,702 were malonyl peptides (Figure 1C) and the modified peptides obtained above contained 1,722 lysine malonylated sites in 810 unique proteins (Figure 1C). All the identified peptides and sites were showed in Supplementary Table 1. The mass spectrograms of three modified peptides were exhibited in Supplementary Figure 1. To validate the mass spectra data, the mass errors of all the malonylated peptides were investigated. It was found that the mass errors of all the identified proteins were less than 5 ppm (Figure 1D), which confirms the MS accuracy fits the experimental requirement (Zhou et al., 2016; Liu et al., 2018). We investigated the molecular weights of all the malonylated proteins obtained from the proteomic data (Supplementary Table 1), and the results showed that the largest protein was acetyl-CoA carboxylase 1 (249.82 kDa, protein accession: A0A1D6GRR8) and the smallest protein was ATP/GTP binding protein (4.23 kDa, protein accession: A0A1D6QMY6). There were 542 modified proteins with molecular weight greater than 25 kDa, which contained 1,254 malonylated sites. The modified protein with molecular weight less than 25 kDa contained 468 malonylated sites in 268 proteins. The molecular weight distribution of these identified proteins was consistent with the immunoblotting signals (Figure 1A). The characteristics of the malonylated proteins were therefore investigated subsequently.

### Analysis of Malonylated Lysine Residues

**FIGURE 2:**
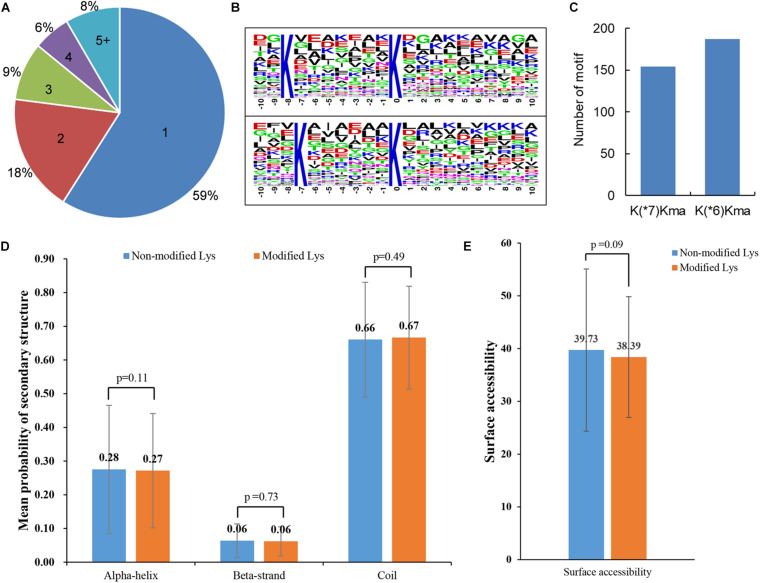
Properties of modified peptides in maize. **(A)** A pie chart of the percentage and number of the modified sites for each identified malonylproteins. **(B)** Enriched sequence motifs containing ± 10 amino acids around the modified sites. **(C)** Number of enriched sequence motifs. **(D)** Mean probability of secondary structure. Wilcoxon test was used to measure the *p*-values. **(E)** Surface accessibility of modified lysine. The *p*-values were calculated with Wilcoxon test.

Protein malonylation may occur on one or more lysine residues. The distribution of all the malonylated sites per protein in maize was therefore calculated. It was found that 478 (59%) identified proteins contained one malonylated site, and the number of malonylated proteins with two, three, four, and five or more modified sites were 146 (18%), 72 (9%), 46 (6%), and 68 (8%) (Figure 2A), respectively. Previous studies have demonstrated that there was a location preference for the modified lysine sites (Zhang et al., 2017; Wang et al., 2019). The amino acid heat map around the modified lysine (from -10 to + 10) was further investigated. The results revealed that lysine (K) appeared most frequently at -7 position, whereas the frequency of serine (S) in position + 6 was the lowest (Supplementary Figure 2). Consistent with the observations in amino acid heat map, two conserved motifs, K(^∗^6)K_ma_ and K(^∗^7)K_ma_ (K_ma_ indicates the malonylated residue and ^∗^ represents an un-specified amino acid) (Figure 2B), were obtained using Motif-x program. As shown in Figure 2C and Supplementary Table 2, the motif K(^∗^6)K_ma_ matched to 187 malonylated peptides, and 154 modified peptides contained the motif K(^∗^7)K_ma_. These observations indicated that the proteins with K in the preference positions were more likely to be modified by malonyl-transferase in maize.

In order to investigate the effects of lysine malonylation on protein structures in maize, a secondary structural analysis of all the malonylated proteins was performed. As shown in Figure 2D, there was no significant difference in secondary structure probabilities (alpha-helix, beta-strand and coil) between modified lysine and un-modified lysine (*p* > 0.1). The absolute surface accessibilities of modified proteins were further investigated, and the results showed that 39.73% of non-malonylated lysine residues were exposed to protein surface, compared with 38.39% of modified lysine sites (*p* = 0.09) (Figure 2E). These observations indicated that lysine malonylation might not affect the surface properties of the malonylated protein.

### Functional Annotation and Intracellular Localization of the Identified Proteins

**FIGURE 3:**
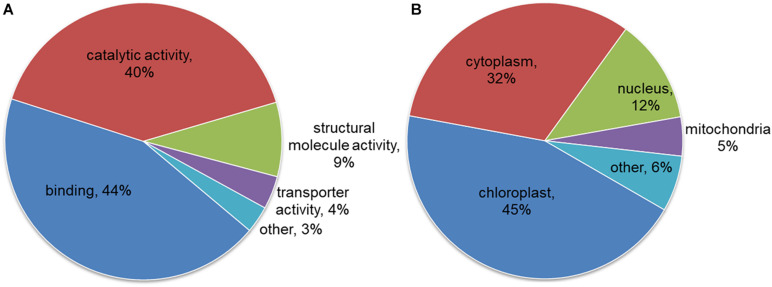
Functional classification for all the modified malonylproteins in maize. **(A)** Classification for the for the modified proteins based on molecular function annotation. **(B)** Analysis the subcellular localizations of the modified proteins in maize.

To better understand the potential role of lysine malonylation in maize, all the modified proteins were classified by GO functional annotation based on their biological processes, cellular components and molecular functions. The results revealed that most of the modified proteins identified from maize were related to metabolic processes (35%), cellular processes (30%) and single-organism processes (19%) according to biological process analysis. The largest proportion of modified proteins were classified to cell (38%) followed by organelle (25%) and macromolecular complex (21%) in term of cellular component annotation. In accordance with these results, a large proportion of the modified proteins were related to binding (44%), catalytic activity (40%) and structural molecule activity (9%) (Figure 3A). The subcellular localizations of the identified proteins were further analyzed, and the result showed that most of the malonylated proteins are located in chloroplast (45%), cytoplasm (32%), nucleus (12%), and mitochondria (5%) (Figure 3B). These observations indicated that the malonylated proteins with diverse functions were widely distributed in maize cells and performed a variety of functions in *Z. mays*.

### Functional Enrichment of the Malonylated Proteins

**FIGURE 4:**
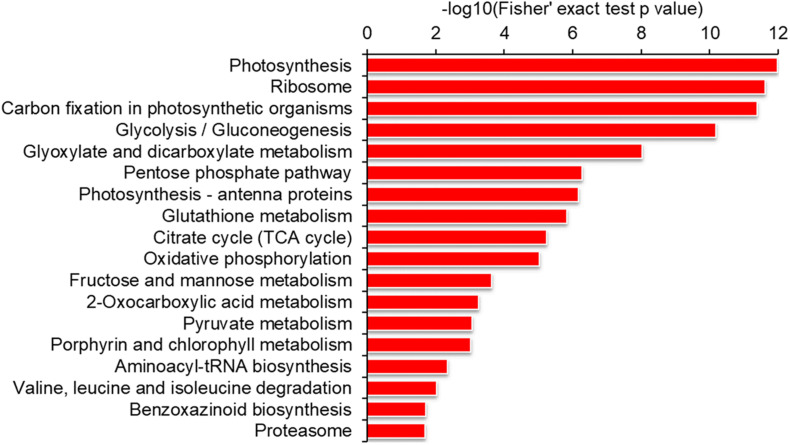
Enrichment analysis of the modified proteins based on KEGG pathway in maize.

To reveal which proteins are susceptible to lysine malonylation, we performed a series of functional enrichment analyses of GO, protein domain and KEGG pathway, respectively. As shown in Supplementary Figure 3 and Supplementary Table 3, the proteins related to structural constituent of ribosome, structural molecule activity, oxidoreductase activity and chlorophyll binding were highly enriched according to GO molecular function enrichment. Consistent with these findings, the proteins located in photosynthetic membrane, thylakoid, chloroplast, cytoplasm and ribosome were significantly enriched based on GO cellular component analysis (Supplementary Figure 3 and Supplementary Table 3). Consistently, the proteins involved in amide biosynthetic process, peptide metabolic process, nucleoside phosphate metabolic process and glycosyl compound biosynthetic process were more likely to be malonylated according to GO biological process enrichment (Supplementary Figure 3 and Supplementary Table 3). In support of these observations, the proteins with domains of NAD(P)-binding domain, chlorophyll a/b binding protein domain, histone-fold, ATPase subunits and ribulose bisphosphate carboxylase large subunit were highly enriched (Supplementary Figure 4 and Supplementary Table 4). Similar observations were also obtained in KEGG pathway enrichment analysis. It was found that a large number of malonylated proteins were highly enriched in photosynthesis, carbon fixation in photosynthetic organisms, glyoxylate, pentose phosphate pathway, citrate cycle, ribosome, glutathione metabolism, oxidative phosphorylation, porphyrin and chlorophyll metabolism (Figure 4 and Supplementary Table 5). Taken together, the malonylated proteins were highly enriched in a variety of protein types and participate in various pathways, indicating that lysine malonylation plays an important role in cell metabolism.

### Analysis of the Malonylated Proteins Related to Photosynthesis and Calvin Cycle

**FIGURE 5:**
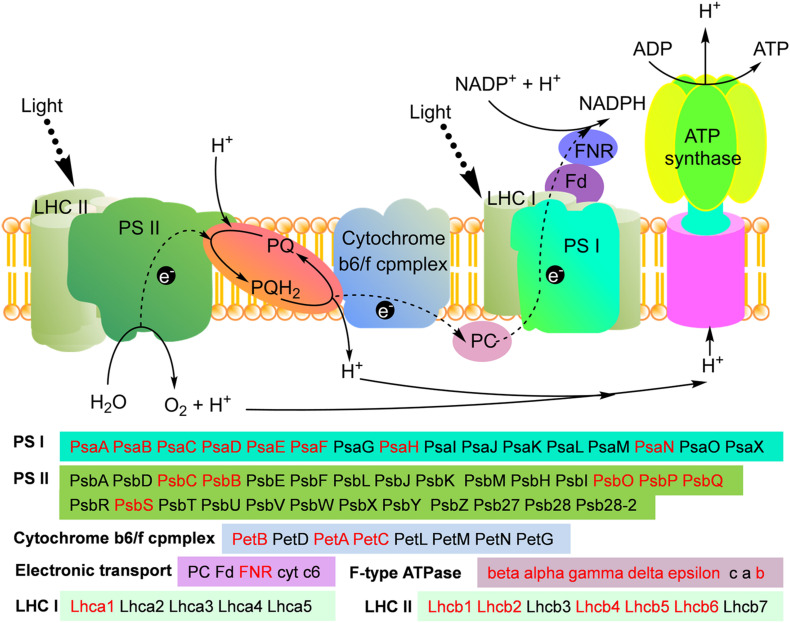
Working scheme of lysine malonylation events associated with in photosynthesis. The identified malonylproteins were marked in red.

Subcellular localization analysis demonstrated that 45% modified proteins were located in chloroplast in maize (Figure 3B). Chloroplast is the site where plant performs photosynthesis. The results of functional enrichment analysis showed that the malonylated proteins associated with photosynthesis and carbon fixation were also significantly enriched (Figure 4). These observations suggest that both photosynthesis and the Calvin cycle may be regulated by protein malonylation in maize. To validate these findings, we obtained the modified proteins involved in photosynthesis and the Calvin cycle in *Z. mays*. Consistent with this hypothesis, a total of 30 malonylated proteins related to photosynthesis were identified in maize (Figure 5), which were distributed in every part of photosynthesis, such as photosystem I (PS I), photosystem II (PS II), cytochrome b6f complex, photosynthetic electron transport, ATPase, and light-harvesting complexes (LHCs) (Figure 5).

**FIGURE 6:**
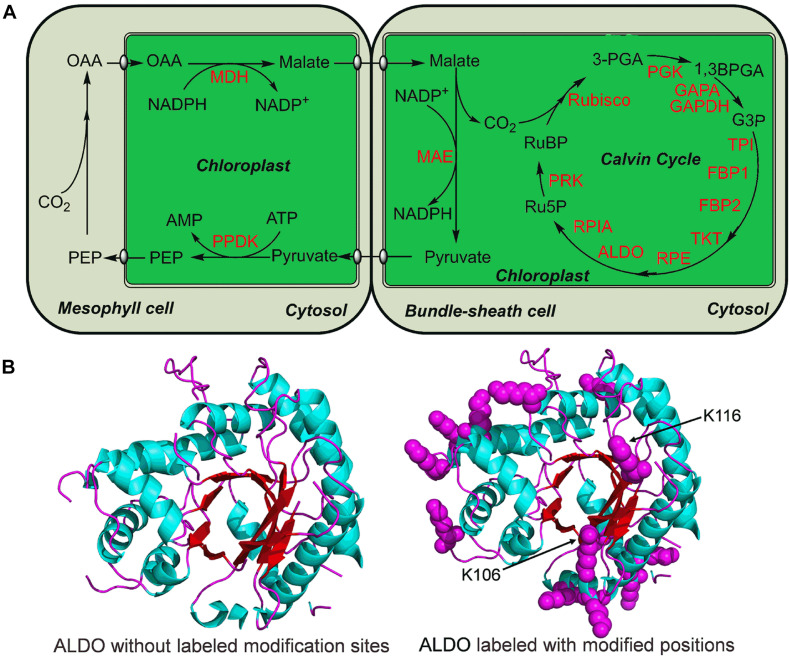
Lysine malonylation enzymes associated with Calvin cycle in maize. **(A)** Reconstruction of Calvin cycle from the KEGG pathway in maize. The modified enzymes were marked in red. **(B)** Three-dimensional structure analysis of ALDO. The malonylated residues were labeled with purple balls. The ALDO three-dimensional structure was modeled from PDB database.

In photosynthesis, carbon dioxide and other compounds are converted to glucose by the Calvin cycle. Maize has a C4 type (C4-dicarboxylic acid) photosynthesis (Figure 6A). It was found that a total of 15 enzymes involved in Calvin cycle were modified by lysine malonylation (Figure 6A). These malonylated enzymes include a large number of key enzymes in Calvin cycle, such as fructose-bisphosphate aldolase (ALDO), ribulose bisphosphatecarboxylase/oxygenase (Rubisco), phosphoglycerate kinase (PGK) and triosephosphate isomerase (TPI) (Figure 6A). Interestingly, ALDO contains 13 malonylated sites in maize (Figure 6B). A 3-dimensional structure of ALDO was constructed, and the results showed that two modification sites, K106 and K116, were located in the catalytic domain of ALDO (Figure 6B). Collectively, these findings support the notion that protein malonylation is involved in regulation of photosynthesis and Calvin cycle in maize.

### PPI Network of the Identified Proteins

**FIGURE 7:**
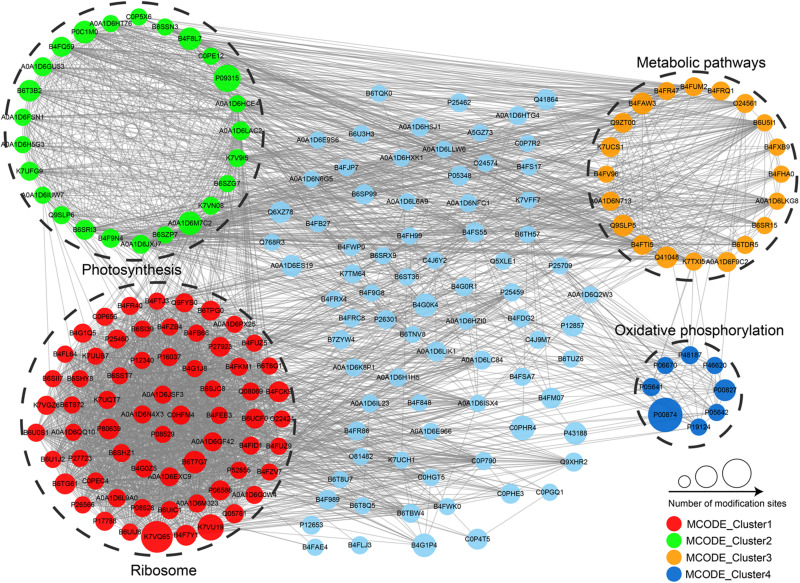
Interaction networks of the identified malonylproteins in maize.

In order to illustrate how these malonylated proteins are involved in multiple metabolic processes, all the identified proteins were searched against the STRING database for PPI network. Finally, a total of 197 modified proteins were mapped to the PPI network database (Figure 7 and Supplementary Table 6), which showed a global view of how the protein malonylation performs various metabolic processes in maize. The top four highly interrelated clusters of malonylated proteins were retrieved. And these top 4 clusters were photosynthesis, ribosome, metabolic pathways and oxidative phosphorylation (Figure 7). The complicated PPI network obtained above indicated that protein malonylation was indeed involved in global cellular regulation in maize.

### Conservative Analysis of the Malonylated Proteins

**FIGURE 8:**
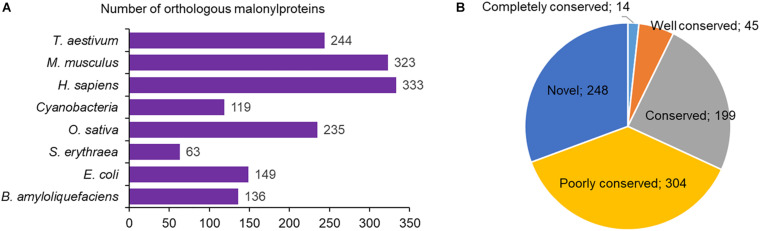
Conservation of the modified malonylproteins. **(A)** Number of orthologous proteins in eight malonylomes reported previously. **(B)** A pie chart illustrating the conservation of malonylated proteins in *H. sapiens*, *B. amyloliquefaciens*, *Cyanobacteria*, *E. coli*, *S. erythraea*, *O. sativa*, *M. musculus*, and *T. aestivum*. The groups were classified as follows: Completely conserved, eight orthologs; Well conserved, six to seven orthologs; Conserved, three to five orthologs; Poorly conserved, one to two orthologs; Novel, zero orthologs.

So far, a large amount of malonylated proteins have been found to be present in both prokaryote and eukaryote. However, compared to the malonylated proteins in maize, we still do not know the conservation of protein malonylation in these species. The conservative analysis of the malonylated proteins was therefore performed. We analyzed the orthologs of the identified proteins in maize by BLAST search against eight malonylomes: *Homo sapiens*, *Bacillus amyloliquefaciens*, *Cyanobacteria*, *Escherichia coli*, *Saccharopolyspora erythraea*, *Oryza sativa*, *Mus musculus*, and *Triticum aestivum*. The results showed that a total of 1,602 orthologous malonylproteins were obtained in the above eight organisms (Figure 8A and Supplementary Table 7). And 562 modified proteins in maize have orthologs in *H. sapiens* (333), *M. musculus* (323), *T. aestivum* (244), *O. sativa* (235), *E. coli* (149), *B. amyloliquefaciens* (136), *Cyanobacteria* (119) and *S. erythraea* (63), which account for 69% (562/810 malonylproteins) of the total modified proteins in maize (Figure 8A and Supplementary Table 7). According to the number of ortholog species, we further classified the conservation of malonyl proteins, and it was found that the number of completely conserved proteins (eight orthologs), well conserved proteins (six to seven orthologs), conserved proteins (three to five orthologs), poorly conserved proteins (one to two orthologous malonylproteins) and novel proteins (zero orthologs) were 14, 45, 199, 304, and 248 (Figure 8B), respectively. These findings indicate that although lysine malonylation is found to be widespread in prokaryotes and eukaryotes, each organism contains its own unique malonylome as a set of malonylated proteins with specific functions.

## Discussion

Lysine malonylation is one of highly conserved PTMs, which has been widely found in both prokaryotes and eukaryotes with multiple functions. The understanding of the role of lysine malonylation in maize, one of the most widely cultivated crops in the world, is still very limited. PTM is a dynamic and reversible process by adding and removing modification groups to proteins. To widely identify the malonylated proteins in maize, we combined the samples from 3 independent biological experiments and performed a proteomics investigation of lysine malonylome in maize using highly sensitive immuno-affinity purification combined with high-resolution LC-MS/MS. Finally, a total of 810 malonylated proteins with 1,722 unique modification sites were identified in maize, including those low-abundance proteins. Similarly, a large number of modified proteins have also been found in plant (Liu et al., 2018) and fungus (Li et al., 2020). These modified malonylproteins are located in diverse subcellular compartments, including nucleus, chloroplast and cytoplasm, belong to multiple functional groups, indicating that lysine malonylation plays an important regulatory role in many cellular processes in maize. The complicated PPI network of the malonylated proteins also demonstrated lysine malonylation as being involved in multiple metabolic processes. To the best of our knowledge, these data represent the first comprehensive view of lysine malonylome in maize.

One of the main physiological metabolisms of maize is photosynthesis, which occurs in chloroplasts. In the chloroplasts, the photosynthesis of maize converts light-energy into chemical energy and stores it in the chemical bonds of sugar. LHCs, the major antennas of photosynthesis (Do et al., 2019; Arsenault et al., 2020), determine how much sunlight can be captured and transferred to the PS I and PS II systems. In maize, one subunit, Lhca1 of LHC I and five subunits of LHC II, Lhcb1, Lhcb2, Lhcb4, Lhcb5, and Lhcb6 were identified to be malonylated (Figure 5). It was found that 4 subunits of LHCs, Lhca1, Lhcb3, Lhcb5 and Lhcb6 were acetylated in common wheat (Zhang et al., 2016). Similar to the results observed in wheat, LHCs of Arabidopsis were also found to be modified by acetylation (Wu et al., 2011). These findings suggest that multiple PTMs, including acetylation and malonylation, may participate in the adjustment of light-harvesting antennas in green plants.

After absorbing sunlight, the photosynthetic systems further convert light-energy into chemical energy and synthesize ATP. In this study, eight subunits of PS I, PsaA, PsaB, PsaC, PsaD, PsaE, PsaF, PsaH, and PsaN and 6 subunits of PS II, PsbC, PsbB, PsbO, PsbP, PsbQ, and PsbS were identified as malonylated proteins in maize (Figure 5). Consistent with our observations, the photosynthetic proteins PsbB, PsbC, PsaA, PsaB, PsaC, and PsaF were malonylated in *Cyanobacteria* (Ma et al., 2017). Photosystems, PS I and PS II, were also found to be modified by acetylation (Zhang et al., 2016) and succinylation (Zhang et al., 2017) in common wheat. We identified several malonylated components in photosynthethic electron transport and ATP synthase, including PetB, PetA, PetC, and PetH in electron transfer and six subunits in ATP synthase (Figure 5). Electron transport chain and ATP synthase can also undergo other PTMs. In Arabidopsis, the b-subunit of chloroplastic ATP synthase was identified as acetylated protein (Wu et al., 2011). Ferredoxin NADP^+^ reductase (FNR), a photosynthethic electron transporter, could be modified by both succinylation (Zhang et al., 2017) and acetylation (Zhang et al., 2016) in wheat. In addition, two electron transporters, FNR and ferredoxin (Fd) were found to be acetylated in cyanobacterium *Synechocystis* sp. PCC 6803 (Mo et al., 2015). Thus, PTMs can be involved in every part of photosynthesis and may regulate their functions in plants and photosynthetic bacteria.

The Calvin Cycle (Calvin-Benson Cycle), takes place in chloroplasts during photosynthesis where carbon dioxide is fixed into sugar. Maize has a C4 dicarboxylic acid pathway in photosynthesis (Pfündel and Meister, 1996). Compared with C3 photosynthesis, C4 photosynthesis has higher light, nitrogen and water use efficiencies (Wang et al., 2014). C-4 plants also have higher cyclic electron-transport activities than C-3 plants (Nakamura et al., 2013). Previous investigations have proved that NADP malic enzyme (MAE) was one of key enzymes in type C4 plants (Nakamura et al., 2013; Nakajima Munekage, 2016). We found that MAE was also modified by malonylation in maize (Figure 6). In photosynthesis, Rubisco is an essential enzyme for photosynthesis (Nagarajan and Gill, 2018), which fixes carbon dioxide into ribulose-1,5-bisphosphate at the first step of the Calvin cycle (Pichersky et al., 1986). As shown in Figure 6, Rubisco was found to be malonylated. Furthermore, ALDO, PGK and glyceraldehyde 3-phosphate dehydrogenase (GAPDH) also underwent lysine malonylation modification (Figure 6). Consistent with these findings, ALDO, PGK, and GAPDH were modified by malonylation in common wheat (Liu et al., 2018). Lysine malonylation participates in the Calvin Cycle indicating its critical role in metabolic regulation of photosynthesis in maize.

## Conclusion

In summary, our results provided the first extensive malonylome dataset in maize. The identified malonylated proteins were related to multiple biological processes, especially in Calvin cycle and photosynthesis. The provided malonylome can be regarded as a rich source for exploring the functions of protein malonylation in maize and likely in all plants.

## Data Availability Statement

The datasets presented in this study can be found in online repositories. The names of the repository/repositories and accession number(s) can be found below: ProteomeXchange, accession no: PXD027417.

## Author Contributions

MX, XT, and TK performed the experiments and analyzed the data. GW and EZ designed the study, analyzed the data, wrote the manuscript, and exercised general supervision. All authors read and approved the final manuscript.

## Conflict of Interest

The authors declare that the research was conducted in the absence of any commercial or financial relationships that could be construed as a potential conflict of interest.

## Publisher’s Note

All claims expressed in this article are solely those of the authors and do not necessarily represent those of their affiliated organizations, or those of the publisher, the editors and the reviewers. Any product that may be evaluated in this article, or claim that may be made by its manufacturer, is not guaranteed or endorsed by the publisher.

## Footnotes

1
http://www.maxquant.org

2
https://www.uniprot.org/

## Abbreviations

AGC: automatic gain control
ALDO: fructose-bisphosphate aldolase
ECL: enhanced chemiluminescence
Fd: ferredoxin
FDR: false discovery rate
FNR: ferredoxin NADP^+^ reductase
GAPDH: glyceraldehyde 3-phosphate dehydrogenase
GO: Gene Ontology
KAT: lysine acetyltransferase
KDACs: lysine deacetylases
KEGG: Kyoto Encyclopedia of Genes and Genomes
LC-MS/MS: liquid chromatography mass spectrometry
LHCs: light-harvesting complexes
MAE: malic enzyme
NCE: normalized collision energy
PGK: phosphoglycerate kinase
PPI: protein-protein interaction
PS I: photosystem I
PS II: photosystem II
PTM: post-translational protein modification
PVDF: polyvinylidene difluoride
Rubisco: ribulose bisphosphatecarboxylase/oxygenase
TPI: triosephosphate isomerase
UPLC: ultra-performance liquid chromatography.
