# Supplementary material for: Global Identification and Systematic Analysis of Lysine Malonylation in Maize (Zea mays L.)
Source: Front Plant Sci. 2021 Aug 20;12:728338. doi: 10.3389/fpls.2021.728338 (PMC8417889; doi:10.3389/fpls.2021.728338)
Supplement: Supplementary file 1 [file Data_Sheet_1.zip › Figure S2.DOC]

**Figure S2.** Amino acids heat map surrounding the malonylated residues
